# Responsiveness to exercise training in juvenile dermatomyositis: a twin case study

**DOI:** 10.1186/1471-2474-11-270

**Published:** 2010-11-25

**Authors:** Clarissa Omori, Danilo ML Prado, Bruno Gualano, Adriana ME Sallum, Ana L Sá-Pinto, Hamilton Roschel, Maria B Perondi, Clovis AA Silva

**Affiliations:** 1 Pediatric Rheumatology Unit, Children's Institute, School of Medicine, University of Sao Paulo (Av. Dr. Arnaldo, 455 - Cerqueira César), Sao Paulo (Postal code: 01246-903), Brazil; 2 School of Physical Education and Sports, University of Sao Paulo (Av Mello de Moaraes, 65 - Butantã), Sao Paulo (Postal code: 05508-030), Brazil; 3 Division of Rheumatology, School of Medicine, University of Sao Paulo (Av. Dr. Arnaldo, 455 - Cerqueira César), Sao Paulo (Postal code: 01246-903), Brazil

## Abstract

**Background:**

Patients with juvenile dermatomyositis (JDM) often present strong exercise intolerance and muscle weakness. However, the role of exercise training in this disease has not been investigated.

**Purpose:**

this longitudinal case study reports on the effects of exercise training on a 7-year-old patient with JDM and on her unaffected monozygotic twin sister, who served as a control.

**Methods:**

Both the patient who was diagnosed with JDM as well as her healthy twin underwent a 16-week exercise training program comprising aerobic and strengthening exercises. We assessed one repetition-maximum (1-RM) leg-press and bench-press strength, balance, mobility and muscle function, blood markers of inflammation and muscle enzymes, aerobic conditioning, and disease activity scores. As a result, the healthy child had an overall greater absolute strength, muscle function and aerobic conditioning compared to her JDM twin pair at baseline and after the trial. However, the twins presented comparable relative improvements in 1-RM bench press, 1-RM leg press, VO_2peak_, and time-to-exhaustion. The healthy child had greater relative increments in low-back strength and handgrip, whereas the child with JDM presented a higher relative increase in ventilatory anaerobic threshold parameters and functional tests. Quality of life, inflammation, muscle damage and disease activity scores remained unchanged.

**Results and Conclusion:**

this was the first report to describe the training response of a patient with non-active JDM following an exercise training regimen. The child with JDM exhibited improved strength, muscle function and aerobic conditioning without presenting an exacerbation of the disease.

## Background

Juvenile dermatomyositis (JDM) is a rare, idiopathic and non-suppurative inflammatory disease that causes proximal muscle weakness and a variety of cutaneous features [1]. Current treatment includes corticosteroids frequently in combination with other immunosuppressive agents such as methotrexate and cyclosporine, or intravenous immunoglobulin. Our group [1-4] and others [5,6] have consistently showed that, despite the improvements in JDM prognosis over the last three decades, a number of patients may develop irreversible cumulative damage due to the disease activity or its treatment.

Patients with JDM often present strong exercise intolerance [6,7]. Furthermore, muscle waste usually occurs during periods of disease activity, leading to muscle weakness [8]. In adults with dermatomyositis and polymyositis [9-12] and inclusion body myositis [13,14], physical training studies have shown improvements in exercise capacity. Therefore, it would be clinically relevant to investigate the effects of exercise training in JDM patients.

This longitudinal case study aimed to investigate the effects of exercise training in a 7-year-old patient with JDM, with her unaffected monozygotic twin sister serving as a control. This approach provided a unique opportunity to evaluate the responsiveness and tolerability to exercise in JDM.

## Methods and Results

A 2-year-old girl presenting muscle weakness, myalgia, difficulty in walking, and re-current episodes of falls, fever and progressive weight loss was first examined at the Pediatric Hospital in 2005. The initial physical evaluation revealed some hematomas, malar and nasal erythema, Gottron's papules on proximal and distal interfalangeans, muscle pain on palpation, proximal muscle weakness (IV - *Medical Research Council*) and the positive Gower's sign. Lactic dehydrogenase [LDH] (563 IU/L, normal range 211-423 IU/L) and aldolase (12.9 IU/L, normal range 1-7.5 IU/L) were increased. Additional biochemical investigations revealed neither systemic inflammation nor increased serum skeletal muscle enzymes (erythrocyte sedimentation rate [ESR]: 16 mm/h, normal range 0-20 mm/h; C-reactive protein [CRP]: 2.1 mg/L, normal range 0-5 mg/L; aspartate aminotransferase [AST]: 23 U/L, normal range 10-34 U/L; alanine aminotransferase [ALT]: 13 IU/L, normal range 10-44 IU/L; creatine kinase [CK]: 60 IU/L, normal range 24-204 IU/L; Antinuclear antibody titres (ANA) and anti-Jo1 were negatives. Esophagogastroduodenoscopy showed a mild gastroesophageal reflux disease and radiographies indicated calcinosis in the right calf, elbows and arms. Echocardiogram, abdominal ultrasound and dual energy x-ray absorptiometry (DEXA) scan revealed no abnormalities. The JDM diagnosis was made according to Bohan and Peter's criteria [15], i.e., the presence of inflammatory infiltrate and perifascicular atrophy at muscle histopathology and typical electromyography abnormalities. Prednisone was promptly initiated in 2005 (1.5 mg/kg/day) and was progressively tapered and then discontinued in 2006. Afterwards, the patient remained in remission without any medication.

According to the parent's report, the children had never been engaged in extra-curricular sports or physical activity programs, even though both of them had been equally encouraged to participate in physical education activities at school. In 2009, the JDM patient (age 7; wt: 23 kg; ht: 1.16 m; VO_2peak_: 41.3 mL/Kg/min) was engaged in a twice-a-week, 16-week combined resistance and aerobic training program. Her unaffected monozygotic twin sister (wt: 23 kg; ht 1.20 m; VO_2peak _42.2 mL/Kg/min) was submitted to the same program, serving as a healthy control. Training sessions consisted of a 5-min treadmill warm-up followed by a 25-min resistance training (bench-press, leg-press, lat-pulldown, leg-extension, and seated-row exercises), a 30-min treadmill aerobic training, and 5-min stretching exercises. Resistance training comprised four sets of 8-12 repetitions-maximum (RM; defined as the maximal load for which a certain number of repetitions can be performed). During the first week, a reduced exercise volume regimen (two sets of 15-20 RM) was adopted. Aerobic training intensity was monitored by a heart-rate (HR) monitor (70% of the HR correspondent to VO_2peak_). All sessions were performed in an intrahospital gymnasium and monitored by two fitness professionals. This study was approved by the local Ethical Committee and informed written consent was given by the subjects' parents.

Prior to and after the 16-week program, we assessed dynamic strength (1-RM for leg press and bench press [16]) and isometric strength (handgrip for the dominant arm and lower back extension). Muscle function was evaluated through timed up-and-go and timed-stands tests [17]. To avoid learning effects, the twins underwent six familiarization sessions, at least 72 h apart, for all strength and functional tests. The coefficients of variation (CV) for these tests were ≤0.5%. The children were also submitted to cardiopulmonary tests to determine the VO_2peak, _ventilatory anaerobic threshold (VAT), exercise time at VAT (tVAT) and time-to-exhaustion, according to a previous description [18]. The physical function was evaluated by the Childhood Health Assessment Questionnaire (CHAQ) [19]. JDM scores included Manual Muscle Test (MMT) [20], Childhood Muscle Assessment Scale (CMAS) [21], Visual Analogue Scale (VAS) and Physician Global Assessment of Disease Activity (PGA) and Parents/Patient Global Assessment of Disease Activity (PaGA) [22]. Laboratory parameters of inflammation and serum skeletal muscle enzymes were also assessed at baseline and 48 h after the last training session.

Except for 1-RM bench press and VO_2peak_, the healthy child tended to present greater absolute strength, function and aerobic conditioning than her twin pair at baseline and after the trial. However, relative improvements (i.e., the percent difference between pre- and post-training) in 1-RM bench press, 1-RM leg press, VO_2peak_, and time-to-exhaustion were similar between the twins. The healthy child presented greater relative increments in lower-back and handgrip strength, whereas the JDM patient presented a higher increase in VAT, tVAT, timed-stands test, and timed-up-and-go test (Table 1).

**Table 1:** Effetcs of a 16-week exercise program on physical capacity in a 7-year-old patient with juvenile dermatomyositis (JDM) patient and in her unaffected monozygotic twin sister.

	Healthy twin			JDM twin		
	
*Variable*	*Pre*	*Post*	Δ *(%)*	*Pre*	*Post*	Δ *(%)*
1RM bench press (Kg)	13.0	17.0	30.8	13.0	17.0	30.8
1RM leg press (Kg)	25.0	30.0	20.0	13.0	15.0	15.4
Handgrip (Kg)	7.0	9.0	28.6	4.0	5.5	37.5
Low-back strength (Kg)	40.0	47.0	17.5	35.0	40	12.5
Timed-stands test (rep)	18.0	19.0	5.5	15.0	18.0	12.0
Timed-up-and-go test (s)	6.6	6.1	-7.6	8.8	6.2	-29.5
VO_2peak _(mL/Kg/min)	42.2	44.7	6.0	41.3	43.6	5.6
Exercise time at VAT (min)	6.0	10.3	71.7	3.0	8.0	166.7
VAT (% VO_2peak_)	80.1	80.5	0.5	57.5	67.8	17.9
VAT (mph)	3.5	3.5	0.0	2.5	3.5	40.0
Time-to-exhaustion (min)	13.3	15.3	15.0	12.3	14.3	16.3

Abbreviations: RM = maximum repetition; VO_2peak _= peak oxygen consumption; VAT = ventilatory anaerobic threshold; Δ = *Delta*.

There was no evidence of exercise-induced exacerbation of inflammation or disease flare. Quality of life scores were also virtually unaltered (Table 2). No clinical evidence of excessive exhaustion, pain, osteoarticular injury, muscle soreness, or any other adverse event was noticed.

**Table 2:** Effects of a 16-week exercise program on clinical and laboratorial parameters related to inflammation, muscle damage, pain, disease activity, and quality of life in a 7-year-old patient with juvenile dermatomyositis (JDM) and in her unaffected monozygotic twin sister.

	Healthy twin		JDM twin	
	
*Variable*	*Pre*	*Post*	*Pre*	*Post*
CMAS	49	49	46	46
MMT	80	80	80	80
CHAQ	0	0	0	0
VAS (Physician's scale)	0	0	0	0
VAS (Parents' scale)	0	0	0	0
PGA	0	0	0	0
PaGA	0	0	0	0
CK (IU/L)	152	201	155	218
LDH (IU/L)	213	166	171	183
Aldolase (IU/L)	6	6	7	7
ESR (mm/hr)	12	15	20	20
C-reactive protein (mg/L)	0.32	0.79	1.73	0.70

Abbreviations: CMAS = Childhood Muscle Assessment Scale; MMT = Manual Muscle Test; CHAQ = Childhood Health Assessment Questionnaire; VAS = Visual Analogic Score; PGA = Physician Global Assessment of Disease Activity; PaGA = Parents/Patient Global Assessment of Disease Activity; CK = Creatine kinase; LDH = Lactate Dehydrogenase; ESR = Erythrocyte Sedimentation Rate. Normal range for CK, LDH, aldolase, ESR and C-reactive protein are 24-192 IU/L, 141-237 IU/L, 1-7.4 IU/L, 0-20 mm/1^st ^hr, 0-5 mg/L, respectively. Score range for CMAS, MMT, CHAQ, PGA and PaGA are 0-52, 0-80, 0-3, 0-10, 0-10, respectively.

## Discussion

JDM is a significant illness that leads to exercise intolerance, disability, and physical inactivity. Since the introduction of new therapies, the attention of outcome measures has shifted from mortality towards morbidity, functional ability, and exercise capacity [1-5,7]. However, no study has investigated the therapeutic role of exercise in JDM. In this context, we reported for the first time the benefits of an exercise training program on physical capacity in a child with JDM. The results obtained were compared with those from her monozygotic unaffected sister, allowing us to assume that the JDM patient is effectively responsive to exercise training.

Notwithstanding the long-term period without disease flare, the JDM child presented overall slightly impaired physical capacity as compared with her healthy peer, corroborating previous observations from Takken et al. [7] who found reduced physical capacity in children with inactive disease *versus *normative healthy values. These findings led the authors to speculate that some exercise parameters lack the responsiveness to improving after an active period of the disease. According to Takken et al. [7], there are several pathophysiologic explanations for the significant impairment in exercise capacity in JDM patients: the increased concentration of intramuscular cytokines, the systemic inflammation process, the inflammation of muscular capillaries, the lack of physical activity, the impaired muscle oxidative capacity, and the effect of glucocorticoid treatment on body mass gain and protein breakdown [7,23]. Interestingly, it has been consistently showed that exercise training counteracts several of these deleterious effects across a variety of diseases, reducing systemic inflammation and vascular reactivity, attenuating glucocorticoid-induced adverse effects, increasing muscle mass and neuromuscular function, reducing fat accumulation, and improving oxidative capacity and neovascularization [24,25]. Because of its vast spectrum of action, exercise has been considered a promising therapeutic tool in JDM [26]. Supporting this concept, the current exercise intervention yielded remarkable improvements in aerobic capacity (e.g., increment of 166.7% in the exercise time at VAT), which is knowingly impaired in JDM patients [7,23].

Historically, exercises and muscle strengthening have often been avoided in JDM. It was believed that exercise may cause muscle fiber damage and inflammation in JDM [24]. However, recent evidence suggests that a single exercise bout for children with non-active JDM does not increase muscle inflammation [27]. Our clinical and laboratory findings further support this concept, suggesting that a controlled exercise program may be tolerable in JDM patients. However, our patient was in remission for approximately three years, thus caution should be exercised in extrapolating these results to patients with active disease.

## Conclusions

This was the first report to describe the training response of a patient with non-active JDM following an exercise training regimen. The child exhibited improved strength, muscle function and aerobic conditioning without experiencing exacerbation of the disease. The clinical observations from this account suggest that this promising intervention merits additional controlled studies.

## Declaration of competing interests

The authors declare that they have no competing interests.

## Authors' contributions

CO, BG and CAAS were responsible for the concept and design; CO, DMLP and BG were significant manuscript writers; AMES, ALSP, HR, BP were responsible for the data acquisition, analysis and interpretation; CAAS was a significant manuscript reviewer. All authors read and approved the final manuscript.

## Consent

Written informed consent was obtained from the patients' relatives for publication of this case report and any accompanying images. A copy of the written consent is available for review by the Editor-in-Chief of this journal.

## Pre-publication history

The pre-publication history for this paper can be accessed here:

http://www.biomedcentral.com/1471-2474/11/270/prepub
